# Knowledge and Practice of Use of Insulin Therapy Among Patients With Type 2 Diabetes Attending Primary Health Care Centers, Riyadh, Saudi Arabia: A Cross-Sectional Study

**DOI:** 10.7759/cureus.35486

**Published:** 2023-02-26

**Authors:** Rasha M Alarfaj, Dalal Alayed

**Affiliations:** 1 Department of Family and Community Medicine, Unaizah College of Medicine and Medical Sciences, Qassim University, Buraydah, SAU

**Keywords:** practice, knowledge, health education, diabetes mellitus, insulin

## Abstract

Objective: The objective of this study was to explore the level of knowledge and practice of insulin therapy among patients with type 2 diabetes in Saudi Arabia.

Materials and methods: In this cross-sectional study, 400 pretested structured questionnaires were administered through an interview with patients in a primary health care center. Responses from 324 participants (81% response rate) were analyzed. The questionnaire comprised three main sections: sociodemographic data, a knowledge assessment, and a practice assessment. The total knowledge score was calculated out of 10: 7-10 was excellent, 5.5-6.9 was satisfactory, and less than 5.5 was poor.

Result: Approximately 57% of the participants were ≤ 59 years old, and 56.3% were females. The mean knowledge score was 6.5 (+/-1.6). Participants showed an overall good practice, with 92.5 rotating the site of injection, 83.3% sterilizing the site, and 95.7% taking insulin regularly. The knowledge level was influenced effectively by gender, marital status, educational level, job, frequency of follow-up, having visited a diabetic educator, duration of insulin therapy, and experiencing a hypoglycemic event (p-value <0.05). Knowledge was revealed to significantly influence self-insulin administration, meal-skipping after taking insulin, use of home glucose monitoring, keeping snacks nearby, and taking insulin in relation to meals (p-value <0.05). In some of the practice parameters, patients with high knowledge scores had better practice.

Conclusion: Knowledge of patients with type 2 diabetes mellitus was satisfactory, with significant differences in knowledge according to gender, marital status, educational level, occupation, duration of diabetes, frequency of follow-up, visiting a diabetic educator, and having an experience of the hypoglycemic episode. Participants showed overall good practice, with better practice being associated with a higher knowledge score.

## Introduction

Type 2 diabetes mellitus (DM) results in the human body's inefficient use of insulin. Out of more than 300 million individuals with DM worldwide, more than 90% have type 2 DM [1,2]. In the middle east, where a high prevalence of DM has been reported, patients often have inadequate knowledge and skills to manage the disease and adequately perform home management [3-5]. DM mortality from direct causes of DM is increasing globally and has reached more than 1.5 million, and more than 80% are in low- and middle-resource countries [6]. 

Insulin is one of the cornerstones of DM treatment. It is vital for patients with type 1 DM and is sometimes required in patients with type 2 DM [7,8]. Insufficient knowledge and improper use of insulin may lead to preventable complications and adverse patient outcomes, which highlights the importance of education as an integral part of a comprehensive DM care program that is often delivered at primary health care centers where more than 90% of patients receive care and treatment for diabetes [7-10]. Studies have been conducted worldwide on the awareness and practice of insulin use in patients with DM, mostly utilizing a survey-based observational approach [11-13].

Few studies conducted in Saudi Arabia have investigated prevalence, complications, and patients with DM's adherence to their medication regimens [14-19]. A survey conducted in Abha, Kingdom of Saudi Arabia, showed that 39.52% of the patients performed self-monitoring of blood glucose; thus, therapeutic management in this country should be improved. Most of them had limited knowledge of insulin usage [20]. 

From 2020 to 2040, a two-fold increase in type 2 DM is expected due to obesity, life expectancy, and the ethnic populations most at risk [21]. An increase is also expected in cardiovascular diseases [22], renal diseases [23], and other long-term DM complications. Hence, lifestyle modifications are highly essential and should be advocated through health education. Therefore, this study aims to explore the level of knowledge and practices regarding the use of insulin therapy among patients with type 2 DM in Saudi Arabia.

## Materials and methods

This cross-sectional study was conducted in three major primary healthcare centers at the National Guard Health Affairs (NGHA). This complex includes the Health Care Specialty Center, King Abdul Aziz City Housing, and National Guard Comprehensive Specialized Clinics. The final sample comprised males and females recruited from the aforementioned locations; they consented to participate, and adult patients with type 2 DM. The further inclusion criteria were on insulin therapy, with or without other hypoglycemic agents, and regularly scheduled follow-ups and regular treatment. The exclusion criteria were individuals who refused to fill in the questionnaire or consent or those unable to present at the health centers for regular follow-ups.

A previously used questionnaire was translated into the Arabic language by two native speakers in English and Arabic [13]. The survey was reviewed by two diabetes consultants and research experts and pretested in a pilot study of 20 participants with similar characteristics to the target population. The questionnaire was self-administered and comprised three main sections: sociodemographic data (seven questions), an assessment of knowledge (10 questions), and an assessment of practice (10 questions) (Appendix A shows the study questionnaire). The total knowledge score was calculated out of 10 and classified according to the score distribution of the pilot as follows: 7-10 was excellent, 5.5-6.9 satisfactory, and less than 5.5 was poor.

The sample size [13,24] calculated was adjusted (with finite population correction) and estimated to be approximately 400. The response rate was 324 participants (81% response rate), who were subsequently analyzed and included in the study. The confidence interval is considered 95%, and the margin error is 5%. The sample size was estimated to be 381 and was adjusted to 400 to compensate for incomplete forms. The sample size was calculated using an open Epi demographic calculator.

Knowledge was scored based on the appropriate response provided by patients with DM to insulin therapy, while practice demonstrated the knowledge of the study population through action. Data were entered by using IBM Corp. Released 2015. IBM SPSS Statistics for Windows, Version 23.0. Armonk, NY: IBM Corp. Descriptive statistics were performed in frequencies and percentages for categorical variables, and mean, and standard deviation (SD) were used for continuous variables. For analytical statistics, the chi-square test (χ2) was used to assess the differences between categorical variables, while one-way ANOVA and t-tests were performed to test differences in continuous variables.

This study's ethical clearance was obtained from the institutional board review (IRB) at King Abdalla International Medical Research Center on April 23, 2017, study number RC17/065/R. Participants were informed of the objectives of the study, and their acceptance was recorded.

## Results

This study was conducted in primary health settings, where the patients with type 2 DM received their routine care according to the health system and care for chronic disease guidelines in Saudi Arabia. The essential findings are regarding the knowledge and practice of the use of insulin. The analyzed sample size was 324 (81% response rate) participants of the 400 participants in the total sample. All the participants were patients with type-2 DM who were using insulin. Their knowledge and practice of insulin use were assessed. The results showed that the mean of the total knowledge score was 6.5 (+/-1.6). The mean knowledge score was higher in male than in female participants and for participants who had the following characteristics: married, educational level, visited a diabetic educator, a long DM duration (>10 years), regular follow-ups of their DM with their primary physician, duration of insulin therapy, and experienced hypoglycemia. Notably, these factors affected the level of knowledge significantly (p-value <0.05) among the studied population. By contrast, the knowledge score did not differ significantly concerning the age of the participants, being on insulin only or insulin and other hypoglycemic agents, and insulin treatment duration (Table 1).

**Table 1:** Mean of total knowledge score according to demographic and general information of study participants (n=324)

	Demographic data	No (%)	Mean knowledge ± SD (0min-10 max)	p-value
Age	59 years or less	186 (57.4)	6.63±1.5	0.55
	60 years or above	138 (42.6)	6.52±1.1	
Gender	Male	142 (43.8)	6.97±1.2	<0.001
	Female	182 (56.2)	6.28±1	
Marital status	Married	264 (79)	6.72±1.9	0.01
	Not married	60 (21)	6.03±2.1	
Educational level	Illiterate	152 (46.9)	6.32±1.5	0.01
	School education	172 (53.4)	6.81±1.04	
Job	Homemaker	127 (39.2)	6.17±1.05	0.01
	Retired	115 (35.5)	6.99±1.2.1	
	Teacher	22 (6.8)	7.1±2.2	
	Military	32 (9.9)	7.03±2.3	
	Other	28 (8.6)	6.5±2.3	
Duration of DM	10 years or less	155 (47.8)	6.42±2.4	0.03
	More than 10 years	169 (52.2)	6.81±3.1	
Current treatment	Insulin therapy only	65 (20.1)	6.73±1.3	0.36
	Mixed therapy	259 (79.9)	6.51±1.5	
Duration of insulin therapy	5 years or less	199 (61.4)	6.45±1.9	0.07
	More than 5 years	125 (38.6)	6.79±1.8	
Frequency of follow-up	Once every month	32 (9.9)	6.03±1.9	0.02
	Once every 3 months	228 (70.4)	6.61±1.5	
	Once every 6 months	58 (17.8)	6.89±1.6	
	Irregular	6 (1.9)	5.16±1.6	
Visited diabetic educator	Yes	207 (63.9)	6.95±1.7	<0.001
	No	117 (36.1)	5.07±1.7	
Experience symptoms of hypoglycemia	Frequent	113 (34.9)	6.9±1.9	<0.001
	Infrequent	171 (52.8)	6.5±1.9	
	Never	40 (12.3)	5.06±1.8	
Satisfaction with insulin administration method	Yes	298 (91.9)	6.65±1.6	0.02
	No	26 (8.1)	5.08±1.6	

The practice of the study participants was tested and compared their knowledge in different aspects. Statistically, ANOVA and the t-test were used to compare the two items (knowledge and practice). The knowledge significantly influenced the practice properly in regards to self-insulin administration, meal-skipping after taking insulin, use of home glucose monitoring, keeping snacks nearby, and the frequency of taking insulin in relation to meals (p-value <0.05; Table 2).

**Table 2:** Distribution according to the comparison of practice with knowledge of participants (n=324)

	Practice assessment items	No. (%)	Mean knowledge ± SD (0min-10 max)	P-value
Do you take insulin yourself or does someone else administer it to you?	Self	252 (77.8)	6.6±1.7	0.02
	Family member	70 (21.6)	6.4±1.3	
	Other	2 (0.6)	3.5±0.7	
Where do you store your insulin?	Refrigerator	315 (97.5)	6.5±1.6	0.18
	At room temperature	9 (2.5)	5.8±1.3	
At what site you prefer insulin to be injected?	Arm	164 (50.6)	6.6±1.6	0.70
	Thigh	114 (35.1)	6.6±1.6	
	Abdomen	46 (14.3)	6.4±1.7	
Do you rotate sites?	Yes	299 (92.5)	6.6±1.6	0.17
	No	25 (7.5)	6.1±1.4	
Do you sterilize the site of injection?	Yes	270 (83.3)	6.6±1.7	0.76
	Sometimes	44 (13.6)	6.5±1.5	
	No	10 (3.1)	6.2±0.9	
When do you take your insulin?	On regular basis	310 (95.7)	6.6±1.6	0.13
	When I feel unwell	14 (4.3)	5.9±2	
Do you skip a meal after taking insulin?	Often	36 (11)	6.1±1.9	0.004
	Sometimes	108 (33.5)	6.2±1.5	
	Never	180 (55.5)	6.8±1.6	
Do you do home gluco-checks?	Regularly	161 (49.6)	6.8±1.6	0.006
	Sometimes	119 (36.8)	6.3±1.6	
	Never	44 (13.6)	6±1.7	
Do you keep a snack in your pocket in case your need it?	Usually	143 (44.2)	6.6±1.5	0.006
	Sometimes	119 (36.7)	6.7±1.6	
	No	62 (19.1)	5.9±1.7	
When do you take your insulin in relation to your meals?	Before meals	215 (66.6)	6.7±1.6	<0.001
	After meals	84 (25.9)	6.4±1.6	
	Not fixed	25 (7.5)	5.2±1.3	

Many participants rotated the injected site (299, 92.3%), stored insulin in a refrigerator (315, 97.2%), sterilized the site of injection (270, 83.3%), kept snacks in case of need and monitored glucose level regularly (143, 44.1%), and regularly checked blood glucose level at home (161, 49.7%). These measures were mostly practiced by the participants who had higher knowledge scores, but some participants practiced despite their lower knowledge scores.

The study population assessed their knowledge related to the use of insulin therapy for type 2 DM. Most participants responded to the question on the multiple types of insulin (188, 58%) correctly, and 137 (42.3%) knew multiple types of insulin delivery devices. Approximately, 233 (71.9%) knew that insulin should not be stopped once the blood sugar level normalizes and that once the insulin started, diet and exercise never became less important, which was correctly answered by 256/324 (79%). Duration of low blood glucose reaction in rapid-acting insulin was known by 152 (46.9%), realizing the right action if just before lunch, and forgot to take insulin at breakfast by 76 (23.5). The other notable responses were as follows: if you take your morning insulin but skip breakfast, your blood glucose level status in the case of taking morning insulin but skipping breakfast (274, 84.6%), signs of hypoglycemia (252, 77.8%), causes of low blood glucose (259, 79.9%), and what to do in case of low blood glucose (307, 94.8%); (Table 3).

**Table 3:** Distribution according to the knowledge assessment among the studied population (n=324)

Knowledge assessment Item	Correct answer	
	No.	%
There are multiple types of insulin (n=324)	188	58
There are multiple types of insulin delivery devices (n=324)	137	42.3
Insulin can be stopped when blood sugar levels normalize (n=324)	233	71.9
Once insulin is started, diet and exercise become less important (n=324)	256	79
Duration of low blood glucose reaction in rapid-acting insulin (n=324)	152	46.9
You realize just before lunch that you forgot to take your insulin at breakfast. What should you do now? (n=324)	76	23.5
If you take your morning insulin but skip breakfast, your blood glucose level will usually be? (n=324)	274	84.6
Not a sign of hypoglycemia (n=324)	252	77.7
Low blood glucose reaction may be caused by too much insulin (n=324)	259	79.9
If you are beginning to have a low blood glucose reaction, you should drink some juice (n=324)	307	94.8

## Discussion

In this study, we explored the knowledge and practice of insulin therapy usage among patients with type 2 DM who attended primary health care centers at NGHA, Riyadh, Saudi Arabia. The study revealed that almost half of the study group either often or sometimes skipped a meal after insulin intake. The importance of this finding is that insulin injection synchronizes with mealtime should be highlighted. More patients in our study were skipping insulin doses or meals after taking insulin than those in studies outside Saudi Arabia [11,13]. The vast majority of this study’s participants stored insulin appropriately; this finding is notable because proper insulin storage results in the appropriate treatment. Regarding the blood glucose monitoring schedule, this study’s respondents showed better results than those in similar studies of Ethiopia and Malaysia [25,26].

More than one-fifth of our study subjects were not self-injecting their insulin, which is markedly increased compared to what is reported in a large-scale Indian study [27]. The abdomen was chosen as the preferred injection site by only 14.3% of our participants, while it was the most commonly preferred site of injection worldwide, as reported by previous studies. Conversely, the arm was selected as the preferred site by 50.6%, while the literature reports rates around 14.4% to 18.8% [27]. The literature has reported that the best means to safeguard healthy tissue is to consistently and adequately rotate the insulin injection sites [28]. According to the recommendations published by the Mayo Clinic, there should be a rotation of injections of the drug. The injection must be at least 1 cm (or approximately the width of an adult finger) from the previous injection site [29]. Moreover, Grassi G et al. reported the association of the technique of the injection with improved glucose control, greater satisfaction with the therapy, better and simpler injection practices, and possibly lower insulin consumption [30].

The overall knowledge of this study’s participants was average, and very few participants scored above the average score. Gender, educational level, occupation, marital status, DM duration, frequency of follow-up, and visiting a diabetic educator were significantly associated with knowledge of insulin use, as lower mean knowledge scores were noted among females, uneducated subjects, homemakers, those with DM for 10 years or less, with insulin therapy for five years or less, having followed up monthly, and not seeing diabetic educator. The same findings were reported by a study conducted in Nepal that assessed DM knowledge in general [31]. Similarly, a survey conducted in the United Arab Emirates showed a significant association between knowledge level and marital status, and educational level, which is consistent with our findings [32]. The synchronization of insulin injection with mealtime must be advocated in any health education opportunity during patients’ care, consultation, or health education sessions.

Although the sample size was adequate for the evaluation, this study's participants were recruited from primary care centers only. Therefore, the findings may not be generalized to the population with DM in the region. Additionally, cross-sectional studies provide a limited amount of strong evidence because the results are based on self-reporting.

## Conclusions

Knowledge of patients with type 2 diabetes mellitus was satisfactory, with significant differences in knowledge according to gender, marital status, educational level, occupation, duration of diabetes, frequency of follow-up, visiting a diabetic educator, and having an experience of the hypoglycemic episode. Participants showed overall good practice, with better practice being associated with a higher knowledge score. The study revealed that in some practice parameters, patients with high knowledge scores had better practices, which indicates the need for educational programs to improve the awareness of the practicing knowledge of insulin therapy. There remains a gap in the knowledge and practice that should be filled with the appropriate counseling and additional patient-centered education. Comprehensive insulin usage educational programs that focus on empowering insulin use among patients with DM and provide DM education and related information as essential elements of DM management programs should be widely available.

## Appendices

Appendix A

Knowledge and practice of the use of insulin therapy among patients with Type 2 diabetes at PHC centers in Riyadh, Saudi Arabia

1. Age __________ years

2. Gender ☐ Male ☐ Female

3. Marital status ☐ single ☐ married ☐ divorced ☐ widowed

4. Occupation ____________________

5. Education ☐ Illiterate ☐ Primary ☐ Secondary ☐ college ☐ others, specify ……………………….

6. How long have you been diagnosed with diabetes?

7. Currently, you are on ☐Insulin only ☐Mixed therapy (insulin and other hypoglycemic agents).

8. How long have you been using insulin?

9. How frequently do you consult a specialist doctor regarding your diabetes?

☐once monthly ☐ every 3 months ☐ every 6 months

☐ others, specify ………. .

10. Did you ever have a session with a diabetic educator? ☐ Yes ☐ No

11. There are many types of Insulin.

☐ True ☐ False ☐ I don’t know

12. There are many types of insulin delivery devices.

☐ True ☐ False ☐ I don’t know

13. Can insulin be stopped once blood sugar levels normalize?

☐yes. ☐ No.

14. Once insulin therapy is initiated, diet and exercise become less important?

i. ☐ Yes ☐ No

15. If you have taken rapid-acting insulin, you are most likely to have a low blood glucose reaction in:

a. Less than 2 hours.

b. 3-5 hours.

c. 6-12 hours.

d. More than 13 hours.

e. I don't know.

16. You realize just before lunch that you forgot to take your insulin at breakfast. What should you do now?

a. Check your blood glucose level to decide how much insulin to take.

b. Skip lunch to lower your blood glucose.

c. Take the same insulin dose that you usually take at breakfast.

d. Take twice as much insulin as you usually take at breakfast.

17. If you take your morning dose of insulin, but you skip breakfast, your blood glucose level will:

a. Increase.

b. Decrease.

c. remain the same.

18. Which of the following is not a sign of hypoglycemia?

a. polyurea.

b. shakiness.

c. sweating.

d. low blood glucose level on a glucometer.

19. A low blood glucose reaction may be caused by:

a. too much insulin.

b. too little insulin.

c. too much food.

d. too little exercise.

20. If you are beginning to have a low blood glucose reaction, you should:

a. drink some juice.

b. do some exercise.

c. lie down and rest.

d. take rapid-acting insulin.

21. Other than from your doctor, from where/whom do you think you can get information regarding insulin use?

☐ Books/Periodicals ☐ Internet ☐ Television ☐ Other sources, specify________ ☐ Not interested in getting the information.

22. How do you take insulin?

☐ Vials ☐ Pens ☐ Both

23. Do you take insulin yourself or does someone else administer it to you?

☐ Self ☐ Family Member ☐ Others, please specify __________

24. Where do you store your insulin?

☐ Refrigerator ☐ at Room Temperature

25. At what site you prefer insulin to be injected?

☐ Arm ☐ thigh ☐ abdomen

26. Do you rotate sites?

☐ Yes ☐ No

27. Do you sterilize the site of injection?

☐ Yes ☐ sometimes ☐ No

28. When do you take your insulin?

☐ regularly ☐ when I feel unwell

29. When do you take your insulin in relation to your meals?

☐ before meals ☐ after meals ☐ not fixed

30. Do you skip a meal after taking insulin?

☐ often ☐ sometimes ☐ never

31. Do you practice home glucose monitoring?

☐ Regularly ☐ sometimes ☐ never

32. Do you carry a snack with you in case you need it?

☐ Usually ☐ sometimes ☐ never

33. Have you ever experienced symptoms of hypoglycemia?

☐ Frequently ☐ sometimes ☐ never

34. Are you confident about insulin self-administration?

☐ yes ☐ No
